# Plant Microbe Interaction—Predicting the Pathogen Internalization Through Stomata Using Computational Neural Network Modeling

**DOI:** 10.3390/foods13233848

**Published:** 2024-11-28

**Authors:** Linze Li, Shakeel Ahmed, Mukhtar Iderawumi Abdulraheem, Fida Hussain, Hao Zhang, Junfeng Wu, Vijaya Raghavan, Lulu Xu, Geng Kuan, Jiandong Hu

**Affiliations:** 1 College of Mechanical and Electrical Engineering, Henan Agricultural University, Zhengzhou 450002, China; lilinze@henau.edu.cn (L.L.); shakeeel.ahmed@stu.henau.edu.cn (S.A.); abdulraheem@stu.henau.edu.cn (M.I.A.); fidahussain@stu.henau.edu.cn (F.H.); hao.zhang@henau.edu.cn (H.Z.); jfwu@henau.edu.cn (J.W.); luluxu@stu.henau.edu.cn (L.X.); gengkuan@stu.henau.edu.cn (G.K.); 2 Henan International Joint Laboratory of Laser Technology in Agriculture Sciences, Zhengzhou 450002, China; 3 Department of Bioresource Engineering, Faculty of Agriculture and Environmental Studies, McGill University, Sainte-Anne-de-Bellevue, QC H9X 3V9, Canada; vijaya.raghavan@mcgill.ca; 4 State Key Laboratory of Wheat and Maize Crop Science, Zhengzhou 450002, China

**Keywords:** plant–pathogen interaction, foodborne illness, foliar water uptake, computational modeling, neural networking

## Abstract

Foodborne disease presents a substantial challenge to researchers, as foliar water intake greatly influences pathogen internalization via stomata. Comprehending plant–pathogen interactions, especially under fluctuating humidity and temperature circumstances, is crucial for formulating ways to prevent pathogen ingress and diminish foodborne hazards. This study introduces a computational model utilizing neural networks to anticipate pathogen internalization via stomata, contrasting with previous research that emphasized biocontrol techniques. Computational modeling assesses the likelihood and duration of internalization for bacterial pathogens such as *Salmonella enterica* (*S. enterica*), considering various environmental factors including humidity and temperature. The estimated likelihood ranges from 0.6200 to 0.8820, while the internalization time varies from 4000 s to 5080 s, assessed at 50% and 100% humidity levels. The difference in internalization time, roughly 1042.73 s shorter at 100% humidity, correlates with a 26.2% increase in the likelihood of internalization, rising from 0.6200 to 0.8820. A neural network model has been developed to quantitatively predict these values, thereby enhancing the understanding of plant–microbe interactions. These methods will aid researchers in understanding plant–pathogen interactions, especially in environments characterized by varying humidity and temperature and are essential for formulating strategies to prevent pathogen ingress and tackle foodborne illnesses within a technologically advanced context.

## 1. Introduction

The cultivation of crops, livestock, and aquatic species occupies over a third of the Earth’s land and ocean areas, impacting global ecosystems and affecting human health and well-being [1,2,3]. Food systems need to evolve to be more sustainable and fair, which necessitates a collaborative approach to knowledge creation across various disciplines [4]. The United Nations Sustainable Development Goals call for food systems to be transformed into more sustainable and equitable models [5]. This transformation involves not only ensuring a stable food supply for all, but also making certain that the processes of food production, distribution, and consumption are ecologically, economically, and socially responsible both now and in the future [6]. Foodborne infections are just one of many potential sources of disease that pose a serious threat to human health around the world impacting the sustainable goal for food systems. According to a recent survey, 420,000 people die each year and one in every ten people become ill as a result of eating pathogen-contaminated food [7,8]. The earliest phase of infection includes the critical process of pathogen penetration into host tissue [9]. Natural surface holes, such as stomata, are important entrance sites for bacterial plant diseases [10,11]. Foliar bacterial plant pathogens are bacteria that infect plants’ leaves and cause a variety of illnesses. These infections can enter the plant via natural openings such as stomata or lesions on the leaf surface. Once inside, they can spread and cause symptoms like spots, blights, and wilts, affecting plant health and crop yields [12,13]. Foliar water uptake contributes significantly to pathogen internalization in plants. According to research, FWU, which allows moisture to be absorbed directly through leaf surfaces, may make stomatal apertures more susceptible to microbial invasion, particularly in humid situations. This is especially relevant for infections *Salmonella enterica* (*S. enterica*), which typically enter through stomata or damaged cuticular layers. Rust pathogens frequently enter through stomata and can alter plant water relations by decreasing guard cell activity, increasing vulnerability to future pathogen attacks [14]. *S. enterica* is a genus of Gram-negative bacteria linked to large global food poisoning outbreaks. It is one of the most frequent foodborne bacteria that cause zoonotic diseases in both terrestrial animals and humans [15,16]. With 93.8 million foodborne diseases and 155,000 fatalities per year, it has emerged as a major global public health concern around the world, putting a strain on the economy due to illness prevention and treatment costs [8,17,18]. Furthermore, studies on endophytic bacteria in crops like lettuce and spinach show that elevated ambient moisture levels associated with Foliar water uptake (FWU) create favorable circumstances for bacterial attachment, survival, and subsequent internalization into plant tissues. This link emphasizes the importance of ambient moisture control and other factors affecting FWU in managing foodborne pathogens in agriculture, especially when accompanied with a complete understanding of plant–microbe interactions [19].

The absorption of water and solutes through stomata by plant leaves has been recognized since more than two centuries [20]. Plant leaf surfaces have microscopic pores called stomata to facilitate gas exchange. The opening and closing of stomata are regulated by guard cells, which respond to various stimuli, including humidity levels and internal plant signals like abscisic acid (ABA), and can serve as entry points for bacteria [21,22]. Bacterial pathogens must first breach plant surfaces to establish infections. Leaf-inhabiting bacteria often exploit natural openings in plant tissues, particularly stomata, to access internal plant cells. While stomata were previously thought to be simple passive entryways, we now understand their role is more complex. Environmental conditions that promote bacterial diseases in leaves, such as elevated humidity and precipitation, may both enhance stomatal opening and create wound sites that provide bacteria alternative routes into plant tissue [10,23].

**Figure 1:**
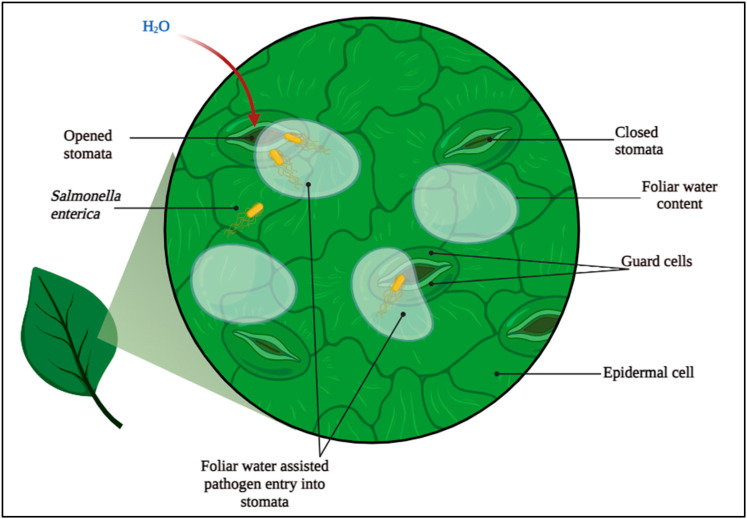
Pathogen insertion linked with foliar water uptake by plants.

While “humidity” describes atmospheric moisture levels, “foliar water content” is a more suitable term when discussing plant–microbe interactions related to bacterial insertion through stomata. However, we adopt the word “humidity” when referring to the leaf surface water content for simplicity. Due to changes in weather conditions, the vapor pressure of the air reaches saturation (or near saturation). Plants can absorb foliar water through their leaves in various ways. When air reaches the saturation point, water condenses into liquid forms like rain [24], dew [25], and fog [26,27] enabling direct leaf absorption. Beyond liquid absorption, research indicates leaves can also take in atmospheric water vapor directly, even without condensation. This vapor absorption process depends on pressure gradients—specifically, when the vapor pressure inside the leaf’s intercellular spaces falls below that of the surrounding air. Such conditions frequently arise because the Kelvin effect and negative water potentials lower the vapor pressure within leaf tissues [28,29]. The findings on FWU’s function in pathogen dynamics provide a foundation for improving food safety through agricultural techniques that reduce pathogen entry in high-humidity situations [30]. The connection between plants and microbes, particularly the bacterial entry through stomata during the process of water absorption, is a complex phenomenon influenced by a variety of environmental factors, including foliar water content, humidity, and leaf surface moisture [11]. Figure 1 captures the scenario of pathogen insertion to plant cells through stomata in the presence of foliar water content.

Current computational advancements in understanding biological processes make it possible to create new models to comprehend the behavior of natural entities [31,32]. These findings are critical to formulate effective strategies to mitigate the risk of foodborne illnesses associated with fresh produce. Keeping this in view, the study presents mathematical models to simulate biological processes in a quantitative way. Parameter identification in multi-parametric, non-linear regression analysis of experimental data requires non-linear biochemical models of dynamic and complex metabolic systems. Because the experimental samples are stochastic, the values that best match the model, the distribution of the parameters, and statistical hypotheses about these values must be estimated. Analytical models for parameter distributions are inadequate because their assumptions are inapplicable to non-linear regressions. Monte Carlo simulations [33] and Partial Differential Equations and Neural Networks [31] serve as a robust instrument for modeling and predicting biological processes.

The designed model takes into account the maximum parameters involved in pathogenic internalization into plants through stomata under different environmental conditions to create comprehensive results. Furthermore, this study employs a neural network-based prediction model to estimate the chance of bacterial penetration through stomata as a function of foliar water content, leading to a greater knowledge of plant–microbe interactions, ultimately benefiting food safety and public health. The paper outlined as in material and method section discusses the physical properties of stomata, model bacterial innate properties, and the environmental factors influencing the pathogenic internalization into plant leaf. Taking into account these physical properties as parameters to model it mathematically leads to the creation of probabilistic modeling and neural networks to predict the bacterial internalization time.

## 2. Material and Method

The following is a step-by-step description of predictive modeling parts and parameters, based on which we can predict the internalization of pathogen under the impact of different environmental effects including foliar water content.

### 2.1. Selection of Basic Parameters

The model is based on the idea that increased foliar water content can enhance stomatal opening due to turgor pressure in guard cells. This condition creates a favorable environment for microbial invasion. Studies indicate that when stomata are open, bacteria can aggregate around these openings and penetrate leaf tissues more effectively [34,35,36]. To design the prediction model for bacterial internalization within plant cells through stomata, we prudently scrutinized innate properties of bacterium and plant leaves’ physical properties to create simulation that closely be similar to natural phenomena [37]. *S. enterica* typically measures 0.7 to 1.5 µm in diameter and 2.0 to 5.0 µm in length [38]. The growth rate of *S. enterica* varies based on environmental conditions, but it has been documented that its maximum growth rate (*μ*_max_) can range from 1.28 to 1.95 h^−1^ under optimal conditions, such as in enteral feeds at around 25 °C [39]. Regarding the diffusion coefficient for *S. enterica* in an aquatic medium, as 0.2 to approximately 1 μm^2^s^−1^ for diameter 1 to approximately 5 μm and velocity up to 39 μms^−1^ [40]. Another study in [41] investigated the diffusion of water and solutes in plant leaves and found that the diffusion coefficient of water was approximately 2.5 × 10^−5^ cm^2^s^−1^, while the diffusion coefficient of solutes was approximately 1.0 × 10^−5^ cm^2^s^−1^. *S. enterica* can survive at a wide range of pH values typically between 5.0 and 7.5 [38,42]. The pH of plant leaves can vary depending on the plant’s growth stage, environmental conditions, and tissue type, generally between 6.0 and 7.5. Stomatal size for leaves ranges from 10 to approximately 80 µm with densities of 5 to approximately 1000 (mm^2^)^−1^, where large size of stomata have less densities [43]. Another study reported stomatal length ranging from 7.699 to approximately 26.914 µm with density ranging from 104.167 to approximately 1069.446 (mm^2^)^−1^ [44].

### 2.2. Designing Mathematical Model

In this section, we present a detailed mathematical framework for simulating the internalization of bacteria through plant stomata under varying environmental conditions. By integrating factors such as humidity, temperature, stomatal size, bacterial motility, and growth rate, we aim to derive a model that accurately predicts the probability and time required for bacterial penetration into the leaf surface. Both deterministic and stochastic methods, including Monte Carlo simulations [33], are employed to account for the inherent randomness in environmental influences and bacterial behavior.

#### 2.2.1. Modeling Internalization Likelihood

Incorporating *(i) Humidity and Stomatal Insertion*, as stated earlier that increased humidity leads to higher water content on the leaf surface, enhancing the mobility of bacteria like *S. enterica*. This increases the chance of bacterial interaction with the stomata. *(ii) Temperature and Stomatal Opening*, Stomatal aperture (opening size) depends on temperature, as stomata tend to open more at specific temperature ranges to facilitate gas exchange. Stomatal opening size influences the probability of bacterial entry. *(iii) Bacterial growth and movement* also depend on temperature, with optimal temperatures enhancing bacterial proliferation and speed.

(a) Impact of Humidity on Bacterial Insertion

To create the mathematical model, we define ‘$P_{insert}$’ as *Probability* of bacterial insertion into stomata, ‘h’ as Relative humidity (in percentage), ‘$D_{h}$’ as Bacterial diffusion coefficient as a function of humidity, ‘$v_{h}$’ will be Bacterial velocity as a function of humidity and ‘$S_{h}$’ as Effective stomatal accessibility area (dependent on humidity and stomatal aperture). Based on the above parameters, the insertion probability $P_{insert}$ can be modeled as follows:

(1) Pinserth=1−e−λ·Dh·AstomaLpore·Tencounterh

where the definitions are as follows:

- $A_{stoma}$: Stomatal area
- $L_{pore}$: Stomatal pore length
- $T_{encounter}\left(h\right)$: Time for bacteria to encounter the stomatal pore, influenced by humidity
- λ: rate constant for pathogen arrival and exponential decay

(b) Humidity Effects on Bacterial Movement

The bacterial diffusion coefficient $D_{h}$ increases with humidity, and it can be modeled as follows:

(2) $$D_{h}=D_{0}\cdot \left(1+\alpha \cdot \left(h-50\right)\right)$$

where the definitions are as follows:

- $D_{0}$: Baseline diffusion coefficient at 50% humidity
- α: Sensitivity coefficient representing how diffusion increases with humidity

Similarly, the *velocity* ‘$v_{h}$’ increases with humidity due to better mobility:

(3) $$v_{h}=v_{0}\cdot \left(1+\beta \cdot \left(h-50\right)\right)$$

where the definitions are as follows:

- $v_{0}$: Baseline bacterial velocity
- β: Sensitivity coefficient for the effect of humidity on velocity

The *time for bacterial encounter* ‘$T_{encounter}\left(h\right)$’ is influenced by both bacterial velocity and diffusion:

(4) Tencounterh=Aleaf22 Dh+Aleaf vh

where the definition is as follows:

- $A_{leaf}$: Area of the leaf where bacteria are moving

Since the bacterial movement is influenced by random environmental conditions, such as varying humidity levels, a Monte Carlo simulation is utilized to simulate bacterial movement and encounter time. By running multiple iterations with randomly generated environmental parameters, we can estimate the probability of distribution of bacterial insertion under real-world conditions. This approach accounts for variability and uncertainties in the diffusion and velocity of bacteria, which are difficult to model deterministically.

(c) Impact of Temperature on Stomatal Opening and Bacterial Growth

The parameters are defined as T: Temperature (in Kelvin or Celsius), $A_{stomata}\left(T\right)$: Stomatal area as a function of temperature, and $\mu_{T}$: Bacterial growth rate as a function of temperature.

The stomatal area $A_{stomata}\left(T\right)$ can be modeled as a Gaussian function of temperature, as stomata tend to open maximally at an optimal temperature $T_{opt}$ and close at extremes as follows:

(5) AstomataT=Amax·e−T−Topt22σT2

where the definitions are as follows:

- $A_{max}$: Maximum stomatal aperture at optimal temperature
- $T_{opt}$: Optimal temperature for stomatal opening
- $\sigma_{T}$: Width of the temperature range where stomata remain open

*Bacterial growth* rate $\mu_{T}$ also follows a temperature-dependent Gaussian model:

(6) μT=μmax·e−T−Topt22σμ2

where the definitions are as follows:

$\mu_{max}$: Maximum bacterial growth rate at optimal temperature
$\sigma_{\mu }$: Width of the temperature range for bacterial growth

(d) Total Insertion Probability under Varied Humidity and Temperature

The combined probability of bacterial insertion is a function of both humidity and temperature as follows:

(7) Pinserth,T=1−e−h·Dh·AstomataTLpore·μT·Tencounterh

This model integrates foliar water content (humidity) and temperature effects on both bacterial movement and stomatal opening. The bacterial insertion probability depends on how well the bacteria can move (diffusion/velocity) and how open the stomata are (temperature effects). By optimizing parameters in this model, we can simulate and predict the likelihood of bacterial internalization under varying environmental conditions.

#### 2.2.2. Model for *S. enterica* Propagation Through Plant Stomata Under Varying Conditions

This model simulates the time required for *S. enterica* to propagate through plant stomata, accounting for variations in pore size, pore length, temperature, and water content. The total time for propagation is influenced by both diffusion and advection (bacterial movement), as well as environmental factors.

The domain represents a cross-section of the plant leaf surface area, with length A=1 mm and discretized into N=100 points with respect to position at dx, given dx=AN, where grid point $x_{i}$ ae equally spaced as xi=A,AN, AN…, A . Taking the bacterial diffusion coefficient, its velocity, stomatal size and length, temperature, and foliar water content, we designed the following model.

The propagation of *S. enterica* is affected by both temperature and humidity. The bacterial movement is modeled as exponentially dependent on temperature as follows:

(8) $$Temp\_effect\left(T\right)=e^{-\left(\;T_{K}-T_{min}/T_{max}-T_{min}\right)}$$

and bacterial velocity and diffusion increase with humidity as follows:

(9) $$Humidity\left(H\right)=1+0.005\cdot \left(H-50\right)$$

The velocity and diffusion are adjusted based on pore size, temperature, and humidity as follows:

(10) $$Adjusted\;Bacterial\;Velocity\;v_{eff}=v\cdot T\cdot H$$

and effective diffusion coefficient $D_{eff}=D\cdot H$. The total propagation time is influenced by two processes i.e., diffusion and advection. The required time for bacteria to propagate by diffusion across the domain is given by the following:

tdiff=A22Deff

whereas time required for bacteria to propagate by advection is,

tadvection=Aveff

Additionally, the running and tumbling phases of bacterial motion are included as 1.25 s for running phase and 0.17 s for tumbling phase.

The *total propagation* ‘$t_{total}$’ is the sum of diffusion, advection, running, and tumbling times as follows:

(11) $$t_{total}=t_{diffusion}+t_{advection}+t_{running}-t_{tumbling}$$

This equation is computed for each combination of pore size, pore length, temperature, and humidity. We first develop a diffusion–advection model to simulate the movement of *S. enterica* through the stomata. Then, using these data, we train a neural network to predict the internalization time under varying environmental and bacterial conditions. The combination of physics-based simulations and machine learning provides a robust approach to understanding and predicting the behavior of the pathogen.

Given that the bacterial propagation time is influenced by several stochastic factors, such as diffusion, advection, and running–tumbling phases, Monte Carlo simulations are employed to generate robust predictions. The random variability in diffusion coefficients, bacterial velocity, and environmental conditions can significantly affect the total propagation time, making Monte Carlo simulations essential for accurately modeling the uncertainty in bacterial internalization.

#### 2.2.3. Predicting Pathogen Internalization Time Using Neural Networks and Diffusion-Advection Models

In the integrated model presented, a FeedForward Neural Network (FFNN) is employed to predict bacterial internalization time based on environmental factors. This neural network configuration facilitates the flow of information in one direction i.e., from input to output, without any cyclic connections. It is particularly effective for tasks such as regression and classification.

Initially, a diffusion–advection model has been developed to simulate the movement of *S. enterica* through the stomata. Then, using these data, we train a neural network to predict the internalization time under varying environmental and bacterial conditions. The combination of physics-based simulations and machine learning provides a robust approach to understanding and predicting the behavior of the pathogen. Data for training neural networks are generated as follows:

The first step in modeling the internalization time of *S. enterica* is to generate synthetic data that capture the effects of environmental factors. In this section, we focus on two primary variables i.e., temperature and humidity, which are known to influence bacterial motility and propagation. Random samples of 1000 temperatures between 20 °C and 32 °C have been generated, keeping humidity between 30% and 100%. These values reflect the environmental conditions that foliar bacteria typically encounter on plant surfaces.

To simulate the movement of bacteria, we use a combination of diffusion and advection dynamics. This model takes into account parameters such as the bacterial velocity, the size of the stomata pores, and the temperature and humidity conditions. The diffusion coefficient and bacterial velocity are adjusted based on environmental effects. This section sets the physical parameters that affect bacterial movement. The diffusion coefficient ‘D’ and the bacterial velocity are key factors in modeling how bacteria traverse the plant surface. The range of pore size and pore length define the size and length of stomata, which varies across plant species.

Monte Carlo simulations were employed to model the combined diffusion and advection dynamics, running thousands of iterations and randomly sampling environmental parameters like temperature, humidity, and pore size. This provides a more realistic prediction of pathogen internalization by estimating the probability distribution of total bacterial propagation time.

For each combination of pore size, pore length, temperature, and humidity, the model calculates the total time taken for bacterial internalization, including the time required for diffusion and advection through the stomatal pore. The defined model calculates the total propagation time based on the combined effects of diffusion and advection. The bacterial velocity and diffusion coefficient are adjusted for the effects of temperature and humidity, ensuring that the model reflects real-world environmental conditions. The results are stored in a 4D matrix (total_time_matrix), capturing the total time for different combinations of pore size, pore length, temperature, and humidity.

#### 2.2.4. Training Neural Network for Prediction

The next step involves using the simulated data to train a neural network that can predict bacterial internalization time given environmental conditions. This network takes as inputs the temperature, humidity, and an additional feature derived from the average propagation time. The internalization time data have been generated and split into training and testing sets. The input data consist of temperature, humidity, and an additional feature that represents the average total propagation time from the simulation. This setup ensures that the neural network has access to relevant environmental variables and the underlying physical properties of the system.

**Figure 2:**
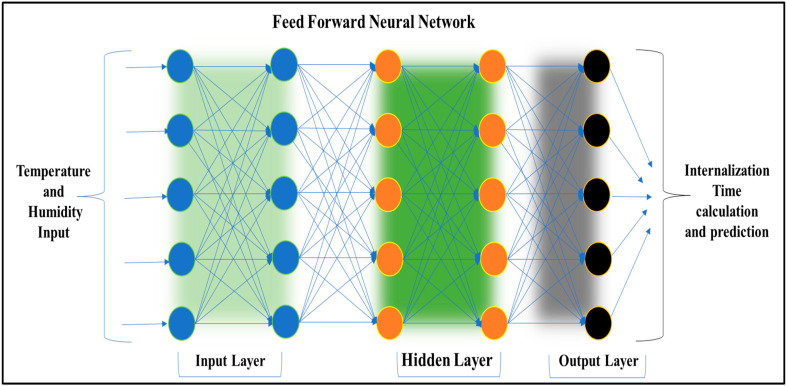
Design of Feed Forward Neural Network.

The neural network is composed of multiple layers to model complex relationships in the data. The input layer, which processes environmental parameters such as temperature and humidity, contains 10 neurons. This is followed by two interconnected hidden layers, each consisting of 5 neurons, which capture intricate patterns and perform internalization calculations. The output layer, containing 5 neurons, predicts the internalization time. The structure depicted in Figure 2 highlights the dense connectivity across layers, demonstrating a robust framework for predictive modeling. The generated dataset was split into three subsets where the first dataset utilizes 70% of the samples for training the network, 20% of the samples for validation to monitor overfitting during training, and 10% of the samples for testing the network’s generalization ability on unseen data. This division aligns with common practices in machine learning and ensures a balanced evaluation of model performance. The training of the network is conducted using the Levenberg–Marquardt algorithm. This method is recognized for its efficiency in training small to medium-sized neural networks, making it especially suitable for regression tasks.

Performance of the neural network is evaluated using the Mean Squared Error (MSE) and the R-squared ($R^{2}$) metric. These metrics provide insight into how well the model predicts internalization times. The ‘$R^{2}$’ value, which ranges from 0 to 1, indicates the proportion of variance in the internalization time that is captured by the model. A value close to 1 suggests a strong predictive ability.

## 3. Results and Discussion

### 3.1. Probabilistic Model of Bacterial Internalization Through Stomata

**Figure 3:**
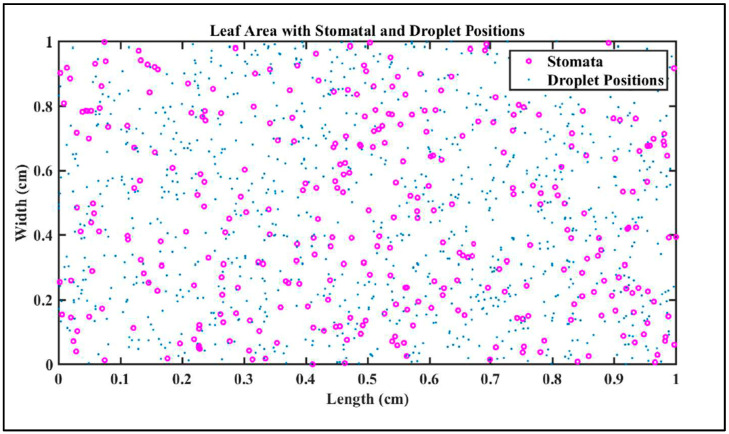
Likelihood of bacterial pathogen internalization ranging from 0.6200 to 0.8820 based on 50% and 100% of humidity levels.

Referring Section 2.2.1, Figure 3 illustrates the probabilistic modeling of bacterial internalization through stomata within a defined leaf area using Monte Carlo simulations [33]. The purple circles represent the random distribution of stomatal sites, which serve as entry portals for bacteria, while the blue dots indicate the positions of droplets containing bacterial agents after dispersion. This model evaluates the migration of bacteria from droplet positions to stomata and calculates the probability of internalization based on key parameters, including stomatal density, bacterial velocity, pore size, and stomatal accessibility.

The simulations incorporate environmental conditions, such as humidity, to assess their impact on bacterial entry. At 50% humidity, the probability of internalization was 0.6200, attributed to reduced stomatal accessibility and limited bacterial mobility. In contrast, at 100% humidity, the probability increased to 0.8820, reflecting enhanced bacterial migration and greater stomatal opening due to higher moisture levels. This approach provides valuable insights into the dynamics of pathogen–stomata interactions under varying environmental and physiological conditions.

### 3.2. Bacterial Pathogen Internalization Time

**Figure 4:**
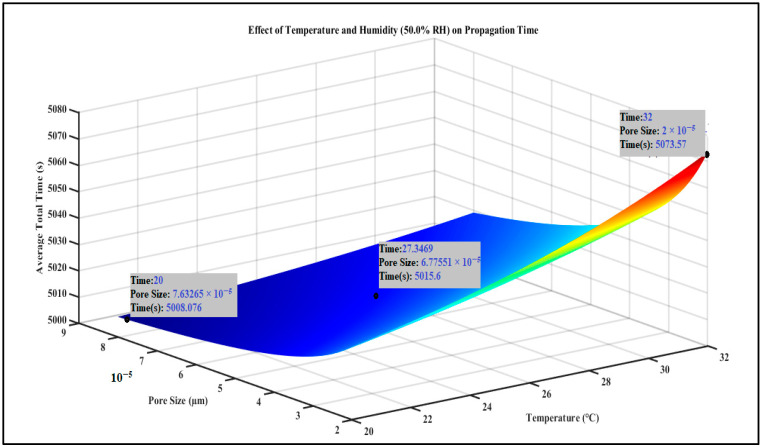
Effect of Temperature and Humidity (50% RH) on Internalization time (Propagation Time) at 50% of humidity, depicting bacteria took more time at lower humidity.

In reference to Section 2.2.2, Figure 4 illustrates the average total time (in seconds) necessary for bacterial internalization as a function of pore size and temperature at 50% relative humidity (RH). The *Z*-axis indicates the cumulative duration of bacterial internalization. The relationship between pore size (μm) on the *X*-axis and temperature (°C) on the *Y*-axis influences the rate of bacterial internalization. The data indicate that increased pore sizes and moderate temperatures (approximately 27 °C) correlate with reduced propagation times (approximately 5000 s), suggesting a more rapid internalization of bacteria. As temperature exceeds 27 °C, the rate of internalization diminishes, resulting in a marginal increase in duration. This relationship may indicate temperature-induced alterations in bacterial motility and stomatal response. The average total propagation time at 50% humidity is 5214.71 s.

**Figure 5:**
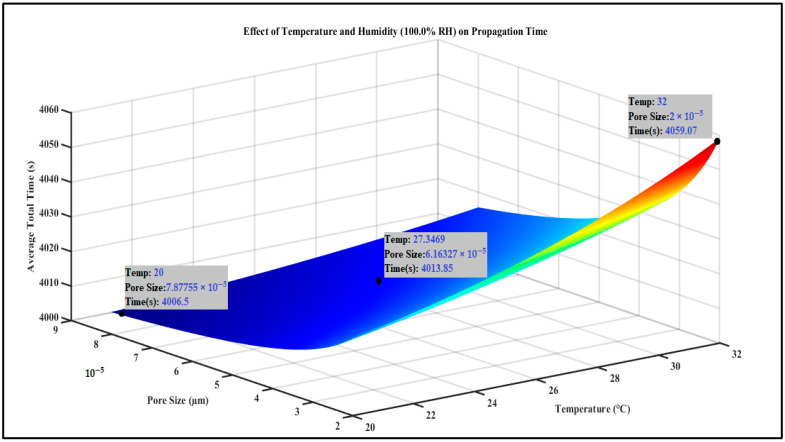
Effect of Temperature and Humidity (100% RH) on Propagation Time.

The plot in Figure 5, akin to Figure 4, assesses the influence of temperature and pore size on the duration needed for internalization, specifically under conditions of 100% relative humidity. Higher humidity (100% RH) results in accelerated internalization across various temperature and pore size ranges, with the minimum time recorded at approximately 4000 s. The *Z*-axis represents the total time for internalization; however, the values are consistently lower compared to the 50% RH scenario. The plot indicates that elevated humidity enhances bacterial movement and penetration of stomata, likely owing to improved moisture conditions that facilitate motility. The optimal pore size and temperature for rapid internalization at 100% relative humidity closely resemble those observed at 50% relative humidity; however, the overall time required is reduced. The average total propagation time at 100% humidity is 4171.98 s. To demonstrate the relationship between the likelihood of internalization and average total propagation time across varying humidity levels, we can examine the impact of propagation time on the probability of successful internalization.

### 3.3. Discussion

The fundamental concept of this study is to provide computational modeling to predict the pathogenic entry into plants through stomata during FWU. The hypothesis is based on the research presented in [12,13] that reduced propagation time correlates with accelerated bacterial movement, potentially enhancing the likelihood of internalization, particularly under elevated humidity conditions that facilitate faster movement of *S. enterica* due to improved moisture availability.

i. Humidity and Speed/Propagation Time: At 50% humidity, the average total propagation time is longer (5214.71 s), which could suggest slower bacterial movement. This results in a lower likelihood of internalization (0.6200). At 100% humidity, the average total propagation time is shorter (4171.98 s), implying faster bacterial movement, which results in a higher likelihood of internalization (0.8820). This aligns with the notion that stomatal dynamics are crucial for regulating pathogen entry and can be influenced by environmental factors such as humidity [45].
ii. Relevance: The data indicate that higher humidity levels (100%) facilitate faster movement of “*S. enterica*”, thereby increasing its chances of reaching and being internalized through stomata. In contrast, lower humidity (50%) results in slower bacterial movement, thereby diminishing its likelihood of internalization within the simulation timeframe [46,47]. This observation underscores the importance of environmental conditions in pathogen dynamics and plant defense mechanisms.
iii. Quantitative Relationship: We can express this relationship using the inverse correlation between likelihood and total propagation time, suggesting the following:

(12) Likelihood of Internalization∝1Propagation Time 

The shorter the propagation time, the higher the likelihood of internalization. The difference in propagation times (around 1042.73 s faster at 100% humidity) corresponds to a 26.2% increase in the likelihood of internalization (from 0.6200 to 0.8820). This shows that higher humidity, which reduces propagation time, can significantly boost the chances of internalization [47,48].

### 3.4. Prediction Through Neural Network Modeling

**Figure 6:**
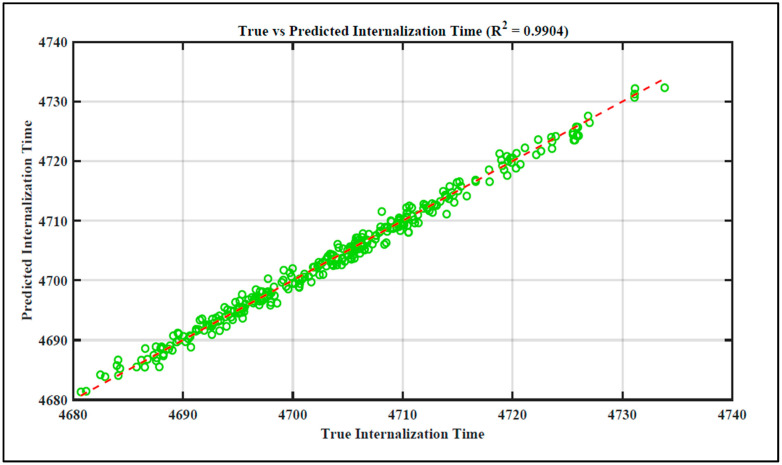
R^2^ for the time range between 4680 s and 4740 s.

The regression plot in Figure 6 shows the predicted versus simulated internalization time (in seconds) for a pathogen’s internalization through stomata, based on our model that includes parameters like temperature, humidity, pore size, and bacterial velocity. The *x*-axis represents the true internalization time (ranging from 4680 s to 4740 s), while the *y*-axis shows the predicted internalization time for the same period.

The regression line closely follows the data points, indicating a strong linear relationship between the true and predicted values. The R^2^ value of 0.9904 shows that 99% of the variance in the true internalization time can be explained by the model’s predictions. This high degree of accuracy suggests that the model effectively captures underlying biological and physical processes influencing pathogen entry [45,49]. This suggests high model accuracy within this time frame, with minimal deviation from the ideal diagonal line (which would represent perfect prediction). The scatter of green circles (representing individual data points) clusters tightly around the red dashed regression line, confirming that the predicted internalization times are highly consistent with the true values. There are very few outliers, demonstrating that the model is effective across the range of internalization times presented (4680 s to 4740 s). The relatively narrow range of internalization times, around 60 s from 4680 s to 4740 s, likely represents a specific environmental scenario where temperature, humidity, or other key factors affecting the pathogen’s internalization process are stable or controlled. This focused range indicates the model’s performance within a specific subset of the entire internalization time spectrum. Figure 6 helps visualize the impact of parameters like temperature and humidity on time internalization. Since the predictions are accurate, it implies that the model has captured the underlying biological and physical processes well. The times in the range of approximately 4680 s to approximately 4740 s could correspond to specific environmental conditions that are conducive to the internalization process.

**Figure 7:**
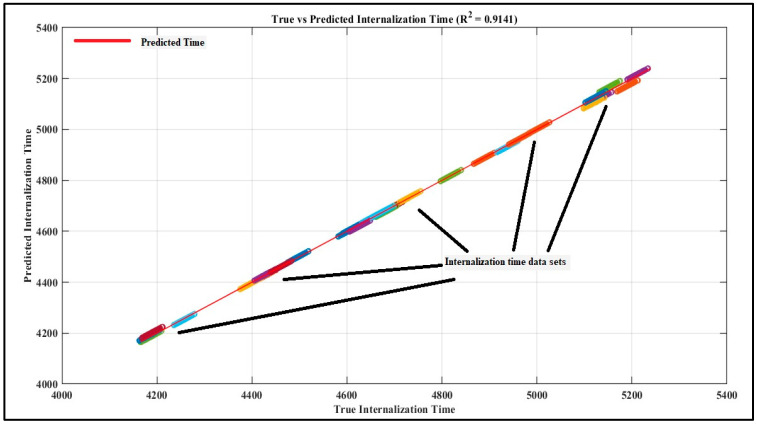
R^2^ value for extended range of simulated time.

We further train the network on a broadened range to capture the maximized variation of the system. The R^2^ value for this was 0.9141 as depicted in Figure 7.

Both plots show a high degree of correlation between the predicted internalization times and the true internalization times, represented by the tight clustering of data points around the red diagonal line (indicating perfect predictions).

Both Figure 6 and Figure 7 confirm a robust correlation between predicted and actual internalization times, evidenced by the tight clustering around the regression line [46,49]. This analysis highlights how environmental parameters like temperature and humidity can significantly affect pathogen internalization times, reinforcing our model’s applicability across different conditions.

The findings of this study emphasize the critical role of environmental factors in pathogen dynamics through stomatal pathways and demonstrate the utility of computational modeling in predicting these interactions effectively. The integration of previous research into our discussion not only strengthens our argument, but also situates our findings within a broader context of plant–pathogen interactions and stomatal physiology [47,48].

### 3.5. Models Predictions

In the following paragraphs, we compare training scenarios of the neural network and its possible impact on the R^2^ value.

(i) Extended Time Range: Figure 6 illustrates that the duration of internalization occurred within a limited interval (i.e., from 4680 to 4740 s). During this period, the model achieved an R^2^ of 0.9904, signifying nearly perfect predictive accuracy. Figure 7 significantly extends the internalization time range, encompassing a broader spectrum from 4000 to 5400 s. The R^2^ value is 0.9141, indicating a good fit, albeit slightly lower than that of the initial plot. The model exhibits a marginal decrease in accuracy for predicting internalization times across the extended time range yet maintains satisfactory overall performance.
(ii) Model Generalization: Figure 6, exhibiting a very high R^2^ value, indicates that the model effectively learnt the relationship within the restricted time frame. Figure 7 demonstrates that the model maintains good generalization across the extended range; however, there is a slight decline in performance, as indicated by a lower R^2^. This suggests the presence of additional variance in the extended data, potentially attributable to non-linear effects at increased internalization times or varying environmental conditions, such as extreme temperatures or humidity.
(iii) Distribution of Points: In Figure 6, the data points exhibit tighter clustering attributed to the restricted time range. The model’s predictions closely matched the true values. In Figure 7, as the time range increases, the data points continue to align with the diagonal trend, albeit exhibiting greater variability and dispersion. This resulted from a more intricate relationship between the features and internalization time at elevated ranges, which the current model is capturing with marginally reduced precision.
(iv) Performance Across Extended Conditions: The model demonstrates a robust linear relationship; however, the variation in R^2^ indicates potential advantages from increased complexity or feature engineering, such as incorporating non-linear interactions among temperature, humidity, and time, to enhance performance under varied environmental conditions or extended internalization durations.

This signifies that the model is capable of accurately predicting internalization times. Both plots depict a linear trend where the predicted internalization times increase as the true internalization times increase, confirming that the model captures the general relationship between the features (temperature, humidity, time) and the internalization time.

## 4. Conclusions

Understanding the relationship between foliar water concentration and bacterial entry through stomata is vital for mitigating foodborne illnesses linked to pathogenic bacteria. The significant global impact of foodborne illnesses underscores the need for innovative approaches to study microbial interactions in plants. Comprehending these interactions can inform agricultural practices aimed at mitigating microbial infections in crops. For different environmental factors and humidity levels, the calculated likelihood is between 0.6200 and approximately 0.8820 and internalization time ranges between 4000 s and 5080 s. Additionally, a neural network model has also been presented to predict these values to enhance the comprehension of plant microbe interactions quantitatively. The calculated R^2^ values for small scaled and wide scaled simulated vs. predicted results are 0.9904 and 0.9141, respectively. Further studies on microbial and environmental regulation of stomatal closure and opening could fill gaps in our consideration of bacterial pathogenesis, disease epidemiology, and microbiology of the phyllosphere. In the future, factors such as leaf surface morphology, light exposure and internal plant stress conditions will also be incorporated in the model that significantly affects bacterial internalization through stomata.

## Data Availability

The original contributions presented in this study are included in the article. Further inquiries can be directed to the corresponding author.
